# Vertebroplasty of C2 Pathologic Fracture: A Unique Case Report Using a Curved-Needle Technique

**DOI:** 10.7759/cureus.25463

**Published:** 2022-05-29

**Authors:** Amar Swarnkar, Sultan Zain, Omari Christie, Kavya Mirchia

**Affiliations:** 1 Neuroradiology, State University of New York Upstate Medical University, Syracuse, USA; 2 Diagnostic Radiology, State University of New York Upstate Medical University, Syracuse, USA

**Keywords:** neuroradiology, vertebral metastasis, cervical vertebroplasty, postero-lateral approach, curved needle, interventional neuroradiology, percutaneous vertebroplasty

## Abstract

Minimally invasive vertebroplasty has arisen as a viable alternative treatment for pathologic vertebral body fractures. Vertebroplasty is well documented in the thoracic and lumbar spine from the posterolateral approach, but is rarely employed in the cervical spine in consideration of numerous critical neural and vascular structures that must be avoided. Careful technique and usage of imaging is necessary to maneuver crucial structures and minimize risk of complication. In the posterolateral approach, the lesion has to lie in the trajectory of a straight needle, in the lateral aspect of the C2 vertebra. This approach may limit adequate treatment of lesions that are located more medially. We describe a unique case report of successful and safe posterolateral approach treatment of a destructive medial C2 vertebra metastatic lesion using a curved needle.

## Introduction

Vertebroplasty involves replacement of the inter-substance of the vertebral body to repair a fracture or structural instability. Cement is typically used as packing material, resulting in; increased vertebral body strength, decreased risk of collapse, and alleviation of pain, especially in patients with osteoporosis or osteolytic bone lesions [1]. Percutaneous vertebroplasty (PVP) is often used in adjunct to analgesics and radiation therapy for pain palliation in patients with vertebral body fractures secondary to malignancy. This procedure is commonly performed in the thoracic and lumbar spine from a posterolateral pedicular or extrapedicular approach. PVP is often not performed in the cervical spine because of the smaller vertebral body size and the technical challenge posed by the presence of vital neurovascular structures within the cervical region such as the spinal cord, carotid artery, jugular vein, and cranial nerves [2]. PVP, specifically at the C2 level, is even rarer because of the anatomic complexity in addition to the relative rarity of neoplastic involvement at the C2 level. In cases of unstable osteolytic lesions, a vertebroplasty can be performed if surgery is considered too challenging. In PVP of a C2 vertebral lesion, a straight needle is typically used from an anterolateral, posterolateral, translateral, or transoral (posterior pharyngeal) approach to avoid critical structures [3]. Use of a straight needle dictates that the lesion must be along that trajectory for adequate treatment. Lesions outside of the direct trajectory may result in limited, inadequate treatment or exclusion from appropriate treatment altogether. Curved-needle PVP technique has been used recently for the lumbar and thoracic spine with reports of increased maneuverability [4,5]. However, the use of the curved needle has not yet been reported in the cervical spine. We describe a case report of posterior approach cervical spine PVP in treatment of rare C2 pathologic fracture secondary to metastatic pancreatic cancer.

## Case presentation

**Figure 1:**
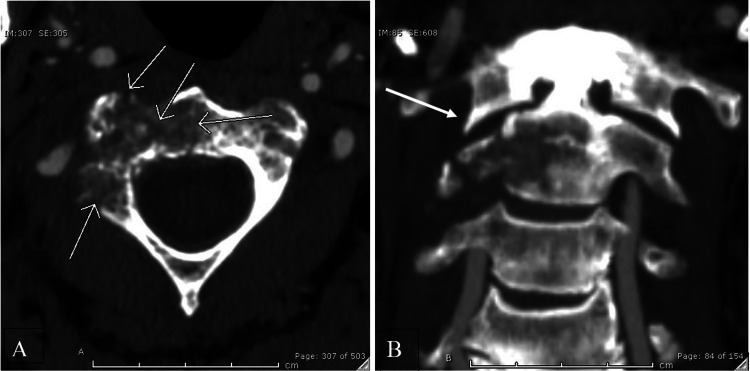
Axial (A) and coronal (B) contrast-enhanced computed tomography (CT) angiogram of the neck depicting the pathologic vertebral body lesion Figure A demonstrates lucency and cortical irregularity (arrows) in the right anterior aspect of the C2 vertebral body. There is asymmetric widening of the right atlanto-axial joint with cortical irregularity at C2 (thick arrow, B). This, along with lucency of the C2 right lateral mass, indicates pathologic fracture.

The patient was a 65-year-old male who presented to the hospital with new onset severe right-sided shoulder and neck pain, unrelieved by over-the-counter medications, for a duration of 10 days. These symptoms were not associated with any numbness or weakness. His past medical history was significant for stage IV metastatic poorly differentiated pancreatic adenocarcinoma, hypertension, and significant alcohol abuse. He had completed six cycles of treatment with FOLFIRINOX (folinic acid/leucovolrin calcium, fluorouracil, irinotecan hydrochloride, and oxaliplatin), but due to progression of disease, had been started on a new regimen with Gemzar and Abraxane two weeks prior. On physical examination, he had no tenderness to palpation over the cervical, thoracic, or lumbar spine. Furthermore, there were no sensory or motor deficits noted in the upper and lower extremities. His reflexes were normal bilaterally. Computed tomography (CT) scan of the cervical spine was performed at an outside hospital, demonstrating osteolytic lesions consistent with metastatic disease involving the right half of the C2 vertebral body, the right lateral mass of C2, adjacent right-side lamina, and depression of the right superior articular surface of the C2 lateral mass (Figure 1). A neurosurgical consultation was placed and, given concern for metastatic lytic lesions, magnetic resonance imaging (MRI) examination of the cervical, thoracic, and lumbar spine was performed. MRI results demonstrated a T2 hyperintense, T1 isointense soft tissue mass lesion replacing the right aspect of the C2 vertebral body, with restricted diffusion and post-contrast enhancement. He was treated with radiotherapy without any significant improvement in the pain. The neurosurgery service recommended no acute surgical intervention. Thus, interventional radiology (IR) was consulted for further management due to significant pain and the risk of instability and ultimately, cord compression. After evaluation, it was decided to proceed with a percutaneous CT-guided C2 vertebroplasty utilizing a posterolateral approach.

Procedure technique

**Figure 2:**
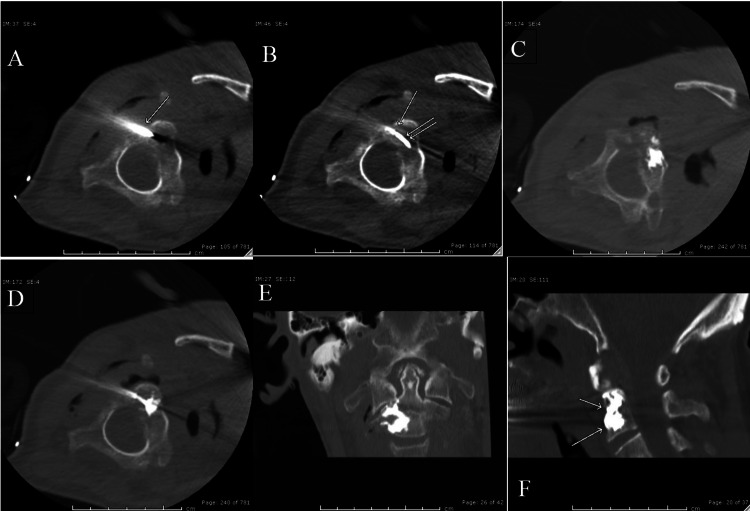
Vertebroplasty intra-procedural images Computed tomography (CT) images depicting the details of procedure. A) Initially, an 11 gauge outer cannula is inserted from the planned right posterolateral approach. B) A curved needle (double arrow) is introduced through the cannula (single arrow) in the lesion. The tip of needle placed further inferiorly and medially. C) Polymethyl methacrylate (PMMA) cement was injected in the inferior portion of the lesion. D) The curved needle was pulled back and reintroduced more superior-medially and further PMMA cement was injected. E) and F) demonstrate post-treatment distribution of PMMA cement in coronal and sagittal planes.

**Figure 3:**
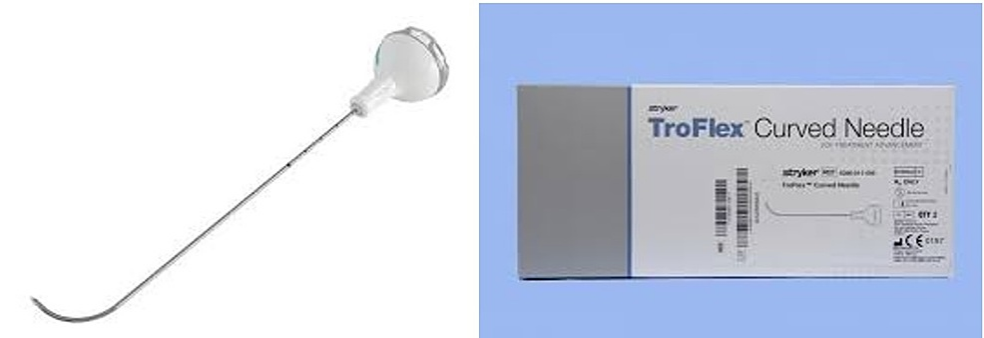
Stryker TroFlex® curved needle system

The patient was placed in the right lateral position and was sedated using 2.5 mg Versed and 125 mcg fentanyl in divided doses. Initially, the C2 vertebral body was localized and 50 cc of IV contrast was given to localize the right vertebral artery and thereby plan the trajectory of approach. Subsequently, from a right posterolateral approach, an 11-gauge introducer needle was advanced into the posterior mid-portion of the vertebral body (Figure 2a). Next, a Stryker TroFlex® curved needle (Figure 3) was inserted and placed into the inferior and medial portion of the osteolytic lesion in the C2 vertebral body (Figure 2b). The polymethyl methacrylate (PMMA) bone cement was prepared as per standard instructions. At this stage, the cement was injected through a curved needle under intermittent CT fluoroscopic guidance (Figure 2c). Once adequate filling of the lower part of the lesion was achieved, the needle was partially withdrawn and rotated to enter the upper paramedian location of the lesion (Figure 2d). There was no resistance to needle repositioning, as this lesion was severely osteolytic. Additional PMMA bone cement was injected in the superior aspect of the lesion. Care was taken to avoid any leakage of cement into the spinal canal or paravertebral soft tissue. Once satisfactory cement filling was achieved, the curved needle was removed. Post-procedural imaging demonstrated successful PMMA cement vertebroplasty (Figures 2e,2f). The post-procedure neurologic exam revealed no deficits. The patient was discharged a few days later with a cervical spine collar. His pain, while not completely resolved, was better controlled. The patient did unfortunately pass away a few months after discharge due to complications of aggressive pancreatic cancer.

## Discussion

Vertebral metastases are most commonly due to breast, prostate, lung, thyroid, renal cell, bladder, and melanotic cancers, with the incidence of skeletal metastases in pancreatic cancer being lower at 5-20% [6,7]. Cervical involvement in pancreatic cancer is even rarer, with only four cases being noted in the literature and no cases involving C2 in particular [8-11]. Vertebral body involvement can be asymptomatic but, when accompanied by fracture, can instead cause uncontrolled pain and instability that is refractory to conservative measures and can predispose patients to cord compression. As such, vertebroplasty is an option utilized to stabilize the vertebra, and has been associated with pain relief in greater than 80% of patients who undergo the procedure [12].

While the procedure can be performed successfully at the C2 level, the complex anatomy poses a technical challenge and, as such, can be subject to complications. There are numerous neurovascular structures passing in close proximity to C2, as it is bounded by the pharynx and larynx anteriorly, the carotid space laterally, the vertebral artery and cervical nerve posterolaterally, and the thecal sac posteriorly [13]. Four approaches are currently used in PVP: anterolateral, posterolateral, transoral, and translateral. The anterolateral approach is generally performed in a supine position and requires hyperextension of the head to elevate the mandible and ease the approach to C2. As such, this technique may not be viable in a patient who cannot maintain hyperextension of the head. The needle is advanced through the parapharyngeal, retropharyngeal, and prevertebral spaces with care taken to manually maneuver the carotid sheath structures posterolaterally. Given this technique, injury to the vertebral artery, carotid artery, jugular vein, submandibular gland, oropharynx, and cranial nerves IX, X, and XI is possible [13]. Cerebellar infarction and C2 neuralgia secondary to cement leakage have also been noted as complications [14]. The posterolateral approach does not require general anesthesia, can be used in patients who are unable to hyperextend their neck, and is generally performed in a prone position. The needle is advanced through the posterior cervical space in an anterior, cranial, and medial direction with care taken to avoid the vertebral artery and the thecal sac. As such, complications involve injury to the vertebral artery and spinal cord [15]. The transoral approach is less technically demanding and involves advancing the needle through the posterior pharyngeal wall and retropharyngeal space. This technique, in addition to possible injury to the vertebral artery, is associated with a higher risk of infection and complications such as retropharyngeal abscess and meningitis. The approach also requires general anesthesia and intubation [13,15]. The translateral approach involves insertion of the needle into a potential space between the carotid sheath and vertebral artery lateral to the C1-C3 level; there is a greater risk of injury to the great vessels in this approach [13]. A complication that can result in any approach is cement leak, which can lead to compression of the spinal cord or nerve roots [16].

The use of a curved needle, as in this case, has been noted to have certain benefits including increased overall flexibility of approach and needle maneuverability. The curved needle facilitates: the ability to selectively target different segments of the vertebral body; more reliable penetration across the midline; shorter procedure duration; lower rate of cement leakage; and reduced fluoroscopy time [4,5]. Based on our review of the literature, the usage of a curved needle at the cervical vertebrae has not been reported, with the aforementioned cases of posterolateral vertebroplasties done at the C2 level utilizing straight needles [15,17-19]. Given the complex anatomy of cervical region, the increased maneuverability of a curved-needle approach may prove to be particularly useful. As demonstrated in our case, the procedure was performed in a comfortable lateral position and we repositioned the needle to fill multiple parts of the lesion. A recent case report from Shah et al. does highlight a retained curved needle after a balloon kyphoplasty, demonstrating a potential complication of curved needles: the shape of the needle may predispose to greater difficulty in removal [20].

## Conclusions

In this case, we demonstrate successful treatment of an unstable pathologic fracture of the C2 vertebra using a posterolateral approach PVP with a curved needle and intermittent CT fluoroscopy, resulting in fracture stabilization and improved pain control. The curved-needle technique was a benefit: it allowed us to reach the lesion from the safer posterolateral approach and gave us the ability to redirect the needle toward various aspects of lesion and adequately and, more completely, fill the lesion defect with PMMA cement. We anticipate that this technique may limit use of anesthesia needed for the transoral pharyngeal approach and avoid neurovascular complications associated with anterior and lateral approaches.
